# Supplementary material for: Disruption of IgA-mediated aggregation at weaning favors mucus encroachment by commensal bacteria
Source: NPJ Biofilms Microbiomes. 2026 Mar 3;12:79. doi: 10.1038/s41522-026-00946-4 (PMC13066159; doi:10.1038/s41522-026-00946-4)
Supplement: Supplementary file 1 — Supplementary Information [file 41522_2026_946_MOESM1_ESM.pdf]

Supplementary Information: "Disruption of IgA-mediated aggregation at weaning favors mucus encroachment by commensal bacteria"

Kevin Simpson[1], Renaud Baillou[1†], Tiphaine Le Roy[2†], Axel Ranson[2], Marta Vazquez-Gomez[2], Delphine Sterlin[3], Guy Gorochov[3], Martin Beaumont[4], Karine Clément[2,5*], Eric Clément[1,6*]

[1]Laboratoire PMMH-ESPCI Paris, PSL Research University, Sorbonne Université and Denis Diderot, Paris, France.
[2]Nutrition and obesities: systemic approaches, Nutriomics, F-75013, Sorbonne University, Inserm, Paris, France.
[3]Centre d'Immunologie et des Maladies Infectieuses (CIMI-Paris), Département d'Immunologie, Hôpital La Pitié-Salpêtrière, AP-HP, Sorbonne Université, Inserm, CNRS, Paris, France.
[4]GenPhySE, Université de Toulouse, INRAE, ENVT, Castanet-Tolosan, France.
[5]Assistance-Publique-Hôpitaux de Paris, Nutrition department, Pitié-Salpêtrière hospital, INSERM, Paris, 75013, France.
[6] Institut Universitaire de France (IUF), Paris France.

*Corresponding author(s). E-mail(s): karine.clement@psl.aphp.fr; eric.clement@upmc.fr;
Contributing authors: kevinsimpson@ug.uchile.cl; baillouaja@gmail.com; tiphaine.le-roy@inserm.fr; axel.ranson@inserm.fr; marta.vazquezgomez@agroparistech.fr; delphine.sterlin@inserm.fr; guy.gorochov@inserm.fr; martin.beaumont@inrae.fr;
†These authors contributed equally to this work.

Supplementary data

Table S1: Statistics for the main text Figure 1e

Contrast	Time	Estimate	SE	df	t.ratio	p.value	signif
cont - test	0	-3.4756	15.52958	16.75205	-0.22381	0.825616	ns
cont - test	2	-10.191	15.52958	16.75205	-0.65623	0.520589	ns
cont - test	4	-19.2096	15.52958	16.75205	-1.23697	0.233159	ns
cont - test	6	-19.2633	15.52958	16.75205	-1.24042	0.231912	ns
cont - test	8	-17.8121	15.52958	16.75205	-1.14698	0.267508	ns
cont - test	10	-25.2359	15.52958	16.75205	-1.62502	0.122822	ns
cont - test	12	-26.275	15.52958	16.75205	-1.69194	0.109174	ns
cont - test	14	-30.8762	15.52958	16.75205	-1.98822	0.063382	ns
cont - test	16	-30.4614	15.52958	16.75205	-1.96151	0.066658	ns
cont - test	18	-40.6413	15.52958	16.75205	-2.61703	0.018186	*
cont - test	20	-40.8009	15.52958	16.75205	-2.6273	0.017805	*
cont - test	22	-45.2921	15.52958	16.75205	-2.9165	0.00973	**
cont - test	24	-47.2123	15.52958	16.75205	-3.04015	0.007488	**
cont - test	26	-53.8156	15.52958	16.75205	-3.46536	0.003012	**
cont - test	28	-50.1198	15.52958	16.75205	-3.22738	0.005022	**
cont - test	30	-54.9198	15.52958	16.75205	-3.53647	0.002584	**
cont - test	32	-52.3845	15.52958	16.75205	-3.37321	0.003672	**
cont - test	34	-57.9909	15.52958	16.75205	-3.73422	0.001687	**
cont - test	36	-60.4345	15.52958	16.75205	-3.89158	0.001202	**
cont - test	38	-61.1668	15.52958	16.75205	-3.93873	0.001086	**
cont - test	40	-61.2528	15.52958	16.75205	-3.94426	0.001073	**
cont - test	42	-68.4598	15.52958	16.75205	-4.40835	0.000397	***
cont - test	44	-71.5307	15.52958	16.75205	-4.60609	0.000261	***
cont - test	46	-70.3044	15.52958	16.75205	-4.52713	0.000308	***
cont - test	48	-71.787	15.52958	16.75205	-4.6226	0.000252	***
cont - test	50	-70.9786	15.52958	16.75205	-4.57054	0.000281	***
cont - test	52	-75.2617	15.52958	16.75205	-4.84634	0.000158	***
cont - test	54	-75.4106	15.52958	16.75205	-4.85593	0.000154	***
cont - test	56	-76.5601	15.52958	16.75205	-4.92995	0.000132	***
cont - test	58	-75.9575	15.52958	16.75205	-4.89115	0.000143	***
cont - test	60	-78.3004	15.52958	16.75205	-5.04201	0.000105	***
cont - test	62	-76.7241	15.52958	16.75205	-4.94051	0.000129	***
cont - test	64	-79.1773	15.52958	16.75205	-5.09848	9.33E-05	****
cont - test	66	-77.9062	15.52958	16.75205	-5.01664	0.000111	***
cont - test	68	-77.4841	15.52958	16.75205	-4.98946	0.000117	***
cont - test	70	-79.2561	15.52958	16.75205	-5.10356	9.23E-05	****
cont - test	72	-79.7562	15.52958	16.75205	-5.13576	8.64E-05	****
cont - test	74	-80.0714	15.52958	16.75205	-5.15606	8.29E-05	****
cont - test	76	-80.6268	15.52958	16.75205	-5.19182	7.7E-05	****
cont - test	78	-80.7596	15.52958	16.75205	-5.20037	7.57E-05	****
cont - test	80	-80.3783	15.52958	16.75205	-5.17582	7.96E-05	****
cont - test	82	-79.9657	15.52958	16.75205	-5.14925	8.4E-05	****

Table S1: Statistics for the main text Figure 1e (Continuation)

Contrast	Time	Estimate	SE	df	t.ratio	p.value	signif
cont - test	84	-81.0969	15.52958	16.75205	-5.22209	7.24E-05	****
cont - test	86	-81.1656	15.52958	16.75205	-5.22651	7.17E-05	****
cont - test	88	-81.1005	15.52958	16.75205	-5.22232	7.23E-05	****
cont - test	90	-80.865	15.52958	16.75205	-5.20716	7.46E-05	****
cont - test	92	-80.5113	15.52958	16.75205	-5.18439	7.82E-05	****
cont - test	94	-81.5704	15.52958	16.75205	-5.25258	6.8E-05	****
cont - test	96	-80.7937	15.52958	16.75205	-5.20257	7.53E-05	****
cont - test	98	-80.1273	15.52958	16.75205	-5.15966	8.23E-05	****
cont - test	100	-79.5621	15.52958	16.75205	-5.12326	8.87E-05	****
cont - test	102	-79.811	15.52958	16.75205	-5.13929	8.58E-05	****
cont - test	104	-80.6662	15.52958	16.75205	-5.19436	7.66E-05	****
cont - test	106	-79.9791	15.52958	16.75205	-5.15012	8.39E-05	****
cont - test	108	-79.8019	15.52958	16.75205	-5.13871	8.59E-05	****
cont - test	110	-79.1715	15.52958	16.75205	-5.09811	9.34E-05	****
cont - test	112	-78.5389	15.52958	16.75205	-5.05738	0.000102	***
cont - test	114	-79.1217	15.52958	16.75205	-5.0949	9.4E-05	****
cont - test	116	-77.7565	15.52958	16.75205	-5.00699	0.000113	***
cont - test	118	-77.5875	15.52958	16.75205	-4.99611	0.000115	***
cont - test	120	-78.14	15.52958	16.75205	-5.03169	0.000107	***
cont - test	122	-77.4989	15.52958	16.75205	-4.9904	0.000117	***
cont - test	124	-76.8447	15.52958	16.75205	-4.94828	0.000127	***
cont - test	126	-76.4769	15.52958	16.75205	-4.9246	0.000134	***
cont - test	128	-76.0418	15.52958	16.75205	-4.89658	0.000142	***
cont - test	130	-75.483	15.52958	16.75205	-4.8606	0.000153	***
cont - test	132	-75.9101	15.52958	16.75205	-4.8881	0.000144	***
cont - test	134	-75.4679	15.52958	16.75205	-4.85962	0.000153	***
cont - test	136	-75.4415	15.52958	16.75205	-4.85792	0.000154	***
cont - test	138	-75.1085	15.52958	16.75205	-4.83648	0.000161	***
cont - test	140	-74.5164	15.52958	16.75205	-4.79835	0.000174	***
cont - test	142	-72.9611	15.52958	16.75205	-4.6982	0.000215	***
cont - test	144	-72.651	15.52958	16.75205	-4.67824	0.000224	***
cont - test	146	-72.7229	15.52958	16.75205	-4.68286	0.000222	***
cont - test	148	-72.9191	15.52958	16.75205	-4.6955	0.000216	***

The significant differences at each time were calculated by the estimated marginal means (EMMeans), corrected by Tukey's method. ns (not significant): $p > 0.05$, $*$: $p \leq 0.05$, $**$: $p \leq 0.01$, $***$: $p \leq 0.001$, $****$: $p \leq 0.0001$.

Table S2: Statistics for the main text Figure 2b

**contrast**	**r/R**	**estimate**	**SE**	**df**	**t.ratio**	**p.value**	**signif**
cont - test	0.1	-1E-05	0.000173	346.4056	-0.05961	0.952504	ns
cont - test	0.2	1.6E-05	0.000129	327.418	0.123916	0.901457	ns
cont - test	0.3	-9.6E-06	0.000119	315.7509	-0.08046	0.935923	ns
cont - test	0.4	-2E-05	0.000115	308.9307	-0.17401	0.861968	ns
cont - test	0.5	-4.1E-05	0.000115	308.9307	-0.35428	0.723368	ns
cont - test	0.6	-0.00012	0.000115	308.9307	-1.01267	0.312011	ns
cont - test	0.7	-0.0003	0.000115	308.9307	-2.63252	0.008901	**
cont - test	0.8	-0.00066	0.000115	308.9307	-5.76685	1.96E-08	****
cont - test	0.9	-0.00126	0.000115	308.9307	-10.9764	6.9E-24	****
cont - test	1	-0.00065	0.000115	308.9307	-5.64801	3.68E-08	****
cont - test	1.1	0.00085	0.000115	308.9307	7.41767	1.16E-12	****
cont - test	1.2	3.37E-05	0.000115	308.9307	0.29433	0.768703	ns
cont - test	1.3	2.85E-06	0.000115	308.9307	0.024874	0.980171	ns
cont - test	1.4	3.02E-05	0.000115	308.9307	0.263859	0.792064	ns
cont - test	1.5	7.32E-05	0.000115	308.9307	0.638761	0.523452	ns
cont - test	1.6	7.46E-05	0.000115	308.9307	0.65067	0.515743	ns
cont - test	1.7	9.04E-05	0.000115	308.9307	0.788366	0.431087	ns
cont - test	1.8	6.52E-05	0.000115	308.9307	0.569092	0.569707	ns
cont - test	1.9	5.94E-05	0.000115	308.9307	0.518402	0.604549	ns
cont - test	2	6.24E-05	0.000115	308.9307	0.54485	0.58625	ns
cont - test	2.1	5.01E-05	0.000115	308.9307	0.437173	0.662291	ns
cont - test	2.2	4.66E-05	0.000115	308.9307	0.40631	0.684796	ns
cont - test	2.3	2.55E-05	0.000115	308.9307	0.222885	0.823772	ns
cont - test	2.4	2.09E-05	0.000115	308.9307	0.182372	0.855411	ns
cont - test	2.5	1.96E-05	0.000115	308.9307	0.171404	0.864019	ns
cont - test	2.6	7.09E-06	0.000115	308.9307	0.061872	0.950705	ns
cont - test	2.7	-1.6E-05	0.000115	308.9307	-0.13648	0.891529	ns
cont - test	2.8	-3E-05	0.000115	308.9307	-0.2584	0.796271	ns
cont - test	2.9	-2.4E-05	0.000115	308.9307	-0.20742	0.835818	ns
cont - test	3	-3E-05	0.000119	315.8225	-0.24787	0.8044	ns

Significant differences at each r/R were calculated by the estimated marginal means (EMMeans), and corrected by Tukey's method. ns (not significant): $p > 0.05$, $**$: $p \leq 0.01$, $****$: $p \leq 0.0001$.

Supplementary figures

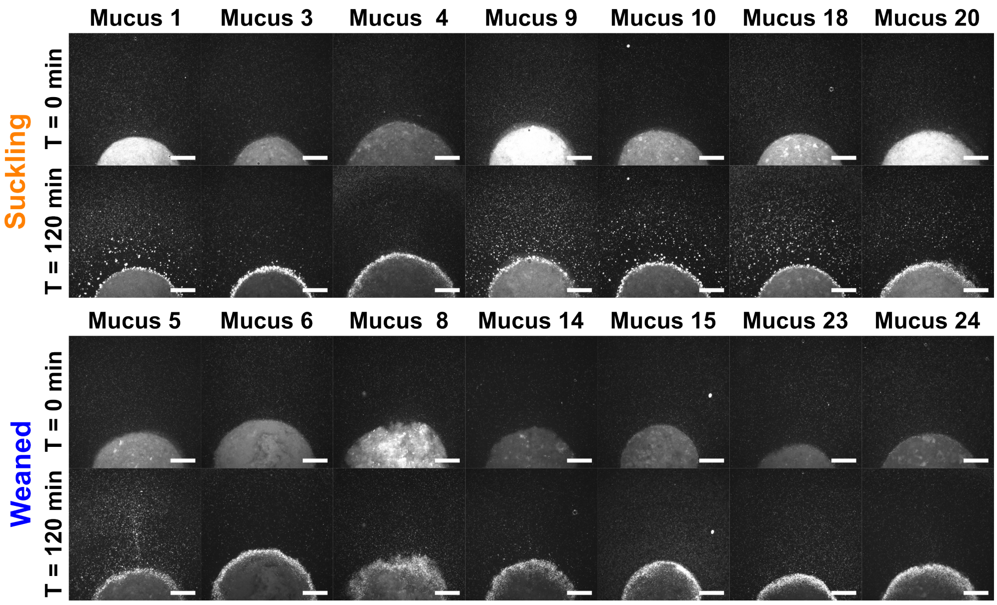

Figure S1 Time-lapse experiments of droplets from the mucus samples used in this work. Images at time-points $T = 0$ min and $T = 120$ min from time-lapse experiments of *E. coli* (bright dots) in a mucus droplet from a suckling or a weaned piglet. The figure shows representative images of droplets from all the mucus samples used in this work to calculate the penetration length and the aggregation of bacteria ($n = 7$ per group). Scale bars 500 μm.

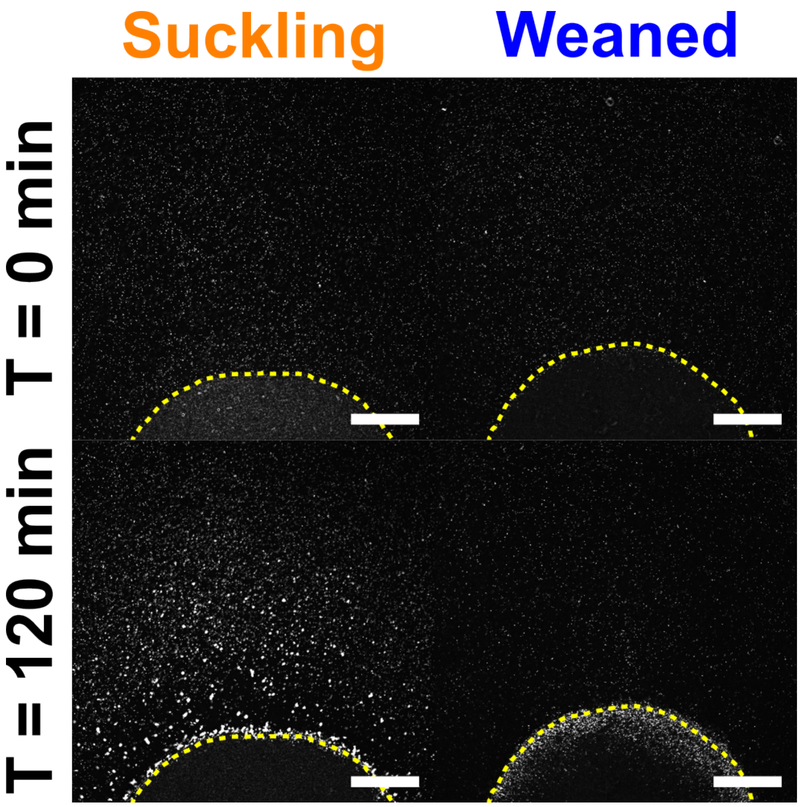

Figure S2 Delimitation of the edges of mucus droplets. First ($T = 0$ min) and last ($T = 120$ min) images of a time-lapse experiment showing the interface between a mucus droplet from suckling and weaned piglets and a bacterial suspension (white dots). The yellow dashed lines represent the edge of the mucus droplets, which was manually defined at $T = 0$ min, and they divide the images into mucus exterior and mucus interior. Scale bars 500 μm.

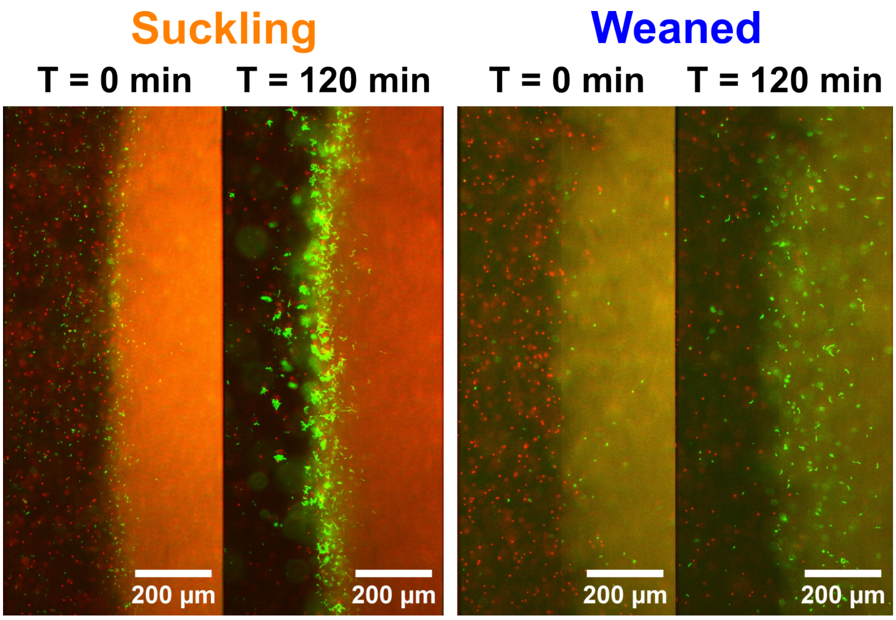

Figure S3 Motility is needed to penetrate the mucus layer. First ($T = 0$ min) and last ($T = 120$ min) images of a time-lapse experiment showing the interface between mucus from suckling and weaned piglets and a bacterial suspension. Passive tracers (1 μm fluorescent beads, red dots) were included in the bacterial suspension to show that they cannot invade the mucus layer whereas the green fluorescent body of motile bacteria can be identified in the mucus layer.

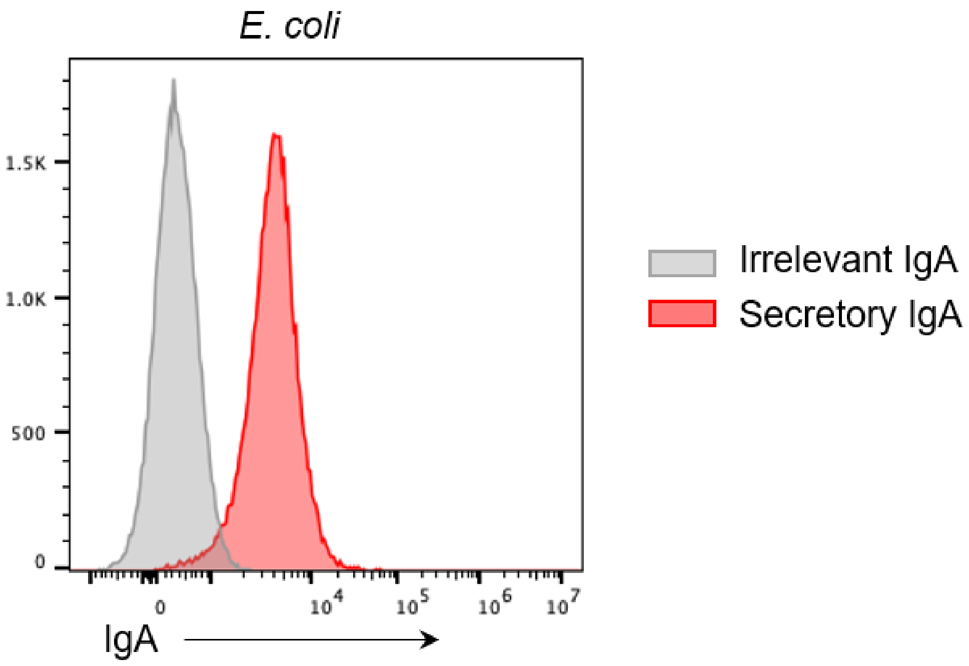

Figure S4 Milk-derived IgA bind the *E. coli* AD62 strain. Representative flow cytometry histogram of purified human breast milk IgA reactivity (red histogram) compared to a human irrelevant anti-KLH IgA (grey histogram) to *E. coli*.

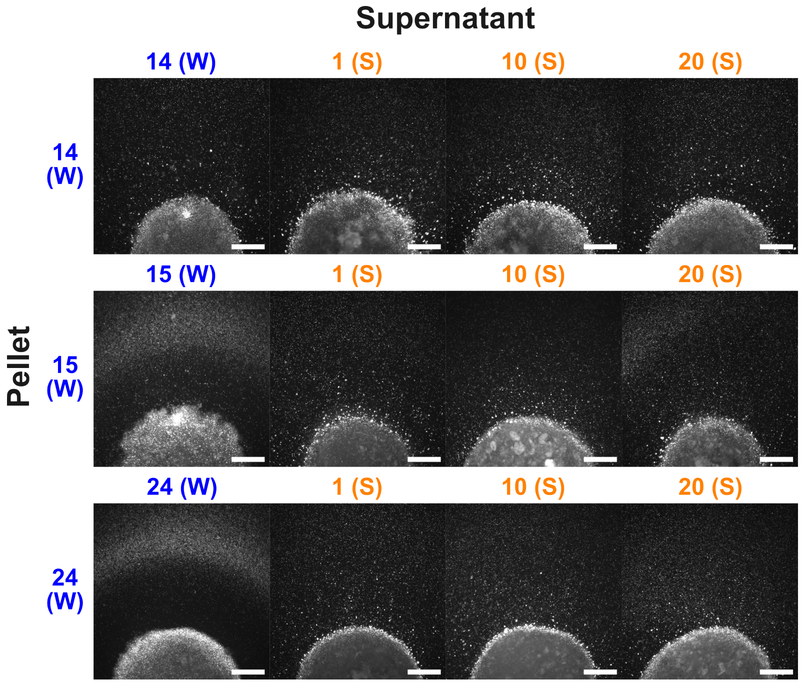

Figure S5 Mucus droplets from weaned piglets supplemented with the supernatant from mucus of suckling piglets. Representative images of mucus droplets obtained by mixing the pellet from the mucus of 3 weaned piglets (e.g., W = weaned, piglets N° 14, 15 and 24) with the supernatant from the mucus of 3 suckling piglets (e.g., S = suckling, piglets N° 1, 10 and 20). Pellet from mucus of weaned piglets mixed with supernatant from mucus of the same weaned piglets was used as controls (W 14,15, 24). Droplets were introduced into the microfluidic system which was then filled with a bacterial suspension, and images were acquired at $T = 120$ min. Scale bars 500 μm.

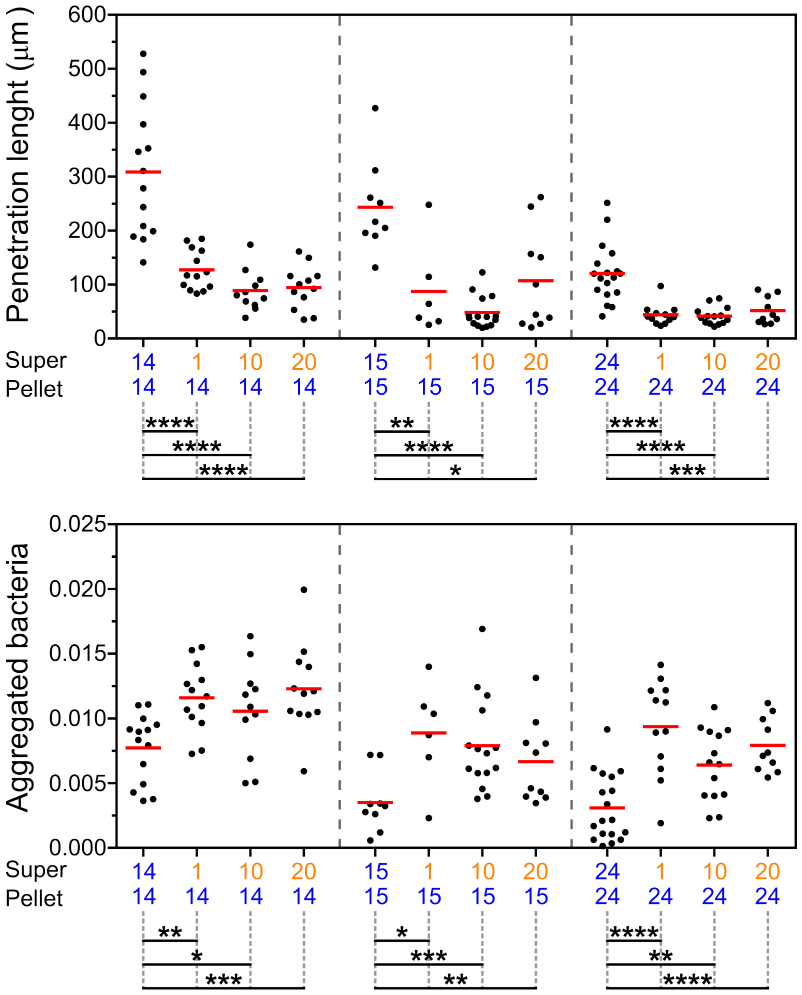

Figure S6 The supernatant from suckling mucus promotes bacterial aggregation and limits bacterial penetration. Quantification of penetration length (top) and total fraction of aggregated bacteria outside mucus (bottom) in mucus droplets composed of different supernatant/pellet combinations. Mucus droplets were obtained by mixing the pellet from the mucus of weaned piglets (e.g., W, piglets N° 14, 15 and 24) with the supernatant from the mucus of suckling piglets (e.g., S, piglets N° 1, 10 and 20). The penetration length was calculated using the data from the interior region, while the mean area and total fraction of aggregated bacteria were calculated for the external region. Blue numbers indicate a pellet or supernatant obtained from the mucus of a weaned piglet, whereas orange numbers indicate a pellet or supernatant obtained from a suckling piglet. Each dot represents and individual droplet. Red horizontal lines denote group means. The table below each graph show the statistical differences based on non-parametric, unpaired two-tailed Mann-Whitney test ($\alpha = 5\%$). $*$: $p \leq 0.05$, $**$: $p \leq 0.01$, $***$: $p \leq 0.001$, $****$: $p \leq 0.0001$. All together, these results indicate that soluble factors present in the mucus supernatant of suckling piglets promote bacterial clustering, producing effects comparable to those observed with purified breast-milk IgA. In contrast, these factors are absent or present at insufficient levels in the supernatant of weaned mucus.

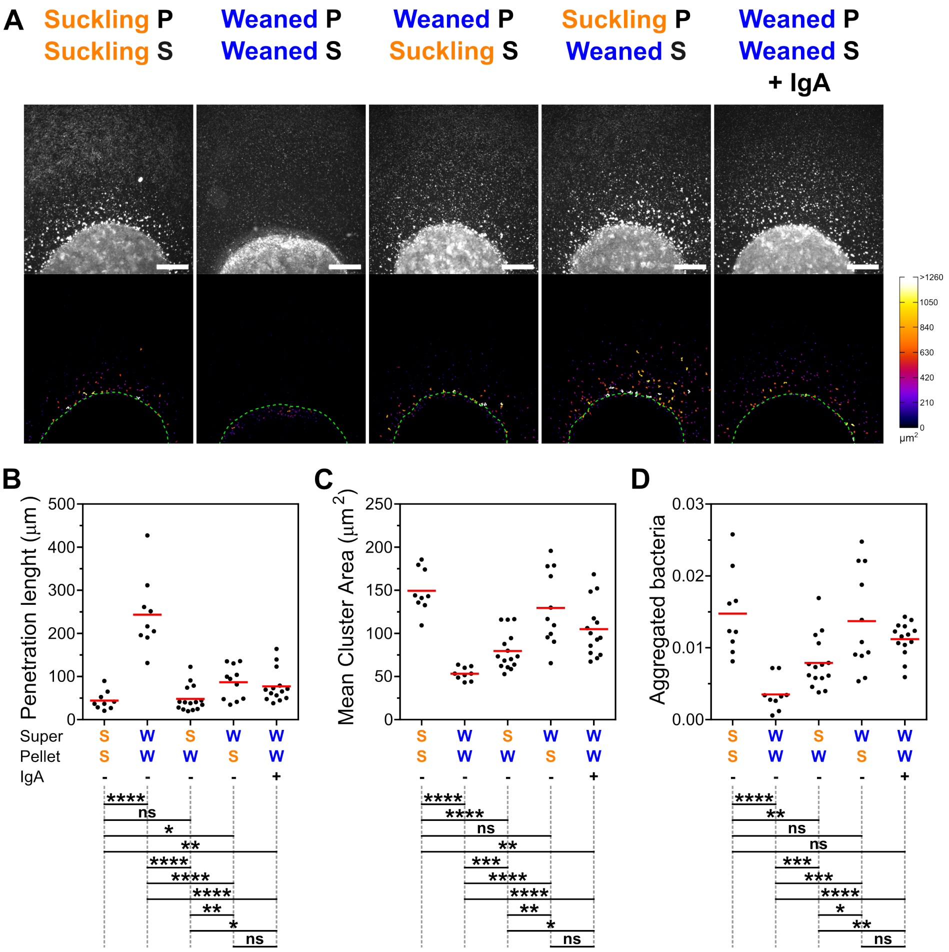

Figure S7 Complete set of supernatant/pellet combinations. (A) Representative images of mucus droplets obtained by mixing supernatant (S) and pellet (P) from suckling and weaned mucus. Droplets were introduced into the microfluidic system which was then filled with a bacterial suspension, and images were acquired at $T = 120$ min. Bottom panels show binarized views of the top panels, with bacterial aggregates color-coded according to their area. Green dashed lines delimitate mucus boundaries. Scale bars 500 μm. (B-D) Quantification of bacterial penetration length (B), mean area of aggregated bacteria (C), and total fraction of aggregated bacteria (D) of mucus droplets composed of different supernatant/pellet combinations from suckling (S) and weaned (W) mucus. The penetration length was calculated using the data from the interior region, while the mean area and total fraction of aggregated bacteria were calculated for the external region. Each dot represents and individual droplet. Red horizontal lines denote group means. The table below each graph show the statistical differences based on non-parametric, unpaired two-tailed Mann-Whitney test ($\alpha = 5\%$). ns (not significant): $p > 0.05$, $*$: $p \leq 0.05$, $**$: $p \leq 0.01$, $***$: $p \leq 0.001$, $****$: $p \leq 0.0001$.

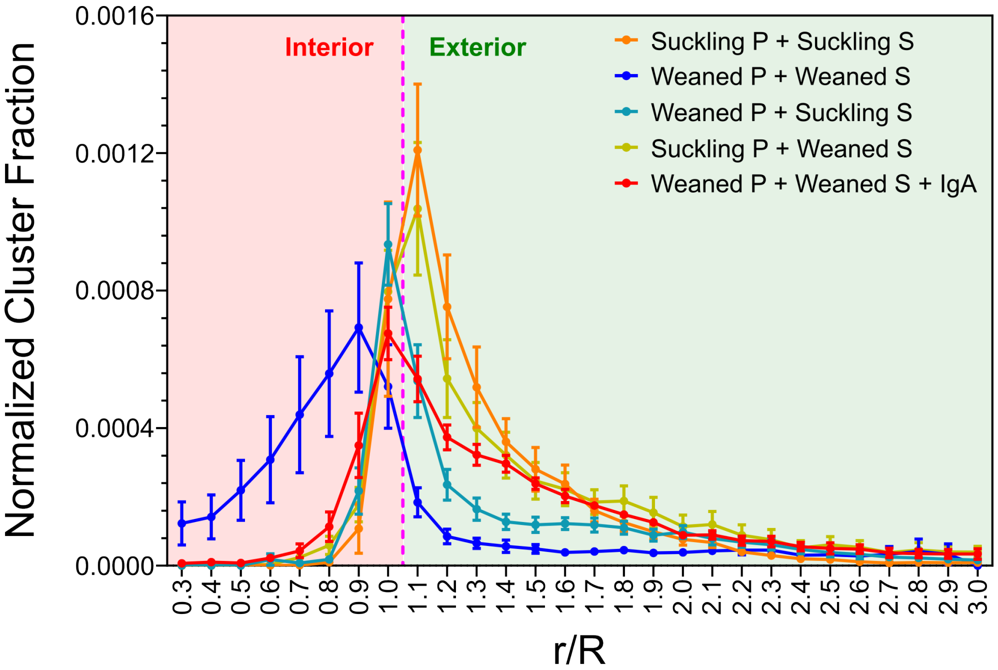

Pair	p value	Significance
Suckling P + Suckling S vs. Suckling P + Weaned S	9.9062E-01	
Suckling P + Suckling S vs. Weaned P + Suckling S	5.0677E-18	****
Suckling P + Suckling S vs. Weaned P + Weaned S	4.1549E-37	****
Suckling P + Suckling S vs. Weaned P + Weaned S + IgA	4.3083E-15	****
Suckling P + Weaned S vs. Weaned P + Suckling S	2.1594E-10	****
Suckling P + Weaned S vs. Weaned P + Weaned S	2.0067E-45	****
Suckling P + Weaned S vs. Weaned P + Weaned S + IgA	4.9252E-08	****
Weaned P + Suckling S vs. Weaned P + Weaned S	4.1162E-34	****
Weaned P + Suckling S vs. Weaned P + Weaned S + IgA	5.7808E-06	****
Weaned P + Weaned S vs. Weaned P + Weaned S + IgA	2.5706E-34	****

Figure S8 Fraction of aggregated bacteria per subregion in supernatant/pellet combinations. Quantification of the normalized fraction of aggregated bacteria at $T = 120$ min across defined subregions of mucus droplets composed of different supernatant (S) and pellet (P) combinations. Data represent the mean $\pm$ standard error of the mean (SEM). Number of replicates: Suckling P + Suckling S: 9, Weaned P + Weaned S: 9, Weaned P + Suckling S: 15, Suckling P + Weaned S: 11, Weaned P + Weaned S + IgA: 14. Magenta dashed line: Mucus boundary. The results of type III ANOVAs performed on linear mixed models derived from these data, 2 groups at 2, are shown below the graph.

Caption of Video_Interface_Suckling_Piglet_1_63X.avi, Movies featuring the aggregation and clustering dynamics of motile *E. coli*, taking place at the mucus/fluid interface for a suckling piglet (piglet #1). The objective magnification is 63X and the movie is accelerated 2X.
